# A Comparative Case Study of Collaborative Governance for Intersectoral Extreme Heat Response in Vancouver, Toronto, and Montreal, Canada

**DOI:** 10.3390/ijerph23040506

**Published:** 2026-04-15

**Authors:** Stephanie Simpson, Mélanie S. S. Seabrook, Erica Di Ruggiero, Lara Gautier, Fiona A. Miller, Monika Roerig, Edward Xie, Sara Allin

**Affiliations:** 1 Institute of Health Policy, Management, and Evaluation, Dalla Lana School of Public Health, University of Toronto, Toronto, ON M5T 3M6, Canada; steph.simpson@utoronto.ca (S.S.); melanie.seabrook@mail.utoronto.ca (M.S.S.S.);; 2 North American Observatory on Health Systems and Policies, University of Toronto, Toronto, ON M5T 3M6, Canada; 3 Social and Behavioural Health Sciences Division, Dalla Lana School of Public Health, University of Toronto, Toronto, ON M5T 3M7, Canada; 4 Department of Health Management, Evaluation and Policy, Université de Montréal, Montreal, QC H3N 1X9, Canada; 5 Collaborative Centre for Climate, Health & Sustainable Care, University of Toronto, Toronto, ON M5T 3M6, Canada; 6 Department of Family and Community Medicine, University of Toronto, Toronto, ON M5G 1V7, Canada

**Keywords:** extreme heat, public health, collaborative governance, intersectoral collaboration, multi-level governance, network governance, health equity, climate change, Canada, comparative case study

## Abstract

**Highlights:**

**Public health relevance—How does this work relate to a public health issue?**

- Extreme heat poses a significant and growing global threat to human health, with disproportionate health impacts experienced by older adults, those of lower socioeconomic status and those with pre-existing health conditions.
- The health impacts of extreme heat may be mitigated through formal heat response plans involving multiple sectors within and beyond government, including public health. This study explores the governance mechanisms shaping intersectoral heat response efforts in Vancouver, Toronto, and Montreal, Canada.

**Public health significance—Why is this work of significance to public health?**

- Through its focus on the social determinants of health, the public health sector may support intersectoral collaboration to effectively and equitably address the negative health impacts of extreme heat.
- Our findings illustrate different roles of public health authorities in intersectoral heat response, including how health equity concerns are addressed.

**Public health implications—What are the key implications or messages for practitioners, policy makers and/or researchers in public health?**

- Public health’s role in extreme heat response is shaped by legislative mandates, provincial guidelines, and formal heat response plans. These may be leveraged to specify a coordinative function for public health authorities to enable the systematic application of an equity lens to intersectoral heat response activities. Sustained funding, dedicated collaborative structures, and informal networks across sectors strengthen intersectoral work.
- Public health researchers and policymakers are encouraged to focus on strategies to improve community-engaged heat response planning, as well as conducting feasible yet robust outcome evaluations of heat response activities.

**Abstract:**

Climate change is an urgent global crisis requiring collaboration across sectors, including public health. In Canada, extreme heat is a leading cause of weather-related mortality, and cities play a central role in mitigating health impacts. This study examined the governance mechanisms shaping intersectoral extreme heat response in Vancouver, Toronto, and Montreal, Canada. Using a comparative case study methodology, we conducted semi-structured interviews (N = 28) and reviewed local heat response documents (N = 30) between November 2023 and December 2024. Thematic analysis informed cross-case comparisons of governance mechanisms shaping collaborative efforts. Across cases, legislative mandates, formal response plans, and coordinating structures for network engagement supported effective intersectoral collaboration. However, collaboration varied in terms of network governance leadership, intersectoral scope (i.e., the type and number of sectors involved), degree of engagement, and the roles of public health authorities. Co-leadership across sectors in Montreal seems to enable greater intersectoral engagement and integration of heat strategies. Areas for improvement include community-engaged heat response planning and enhanced capacity for conducting heat response outcome evaluations. Public health authorities may inform the strategic direction of future heat strategies by supporting the application of a population health lens and facilitating intersectoral collaboration to better address the upstream determinants of heat health inequities.

## 1. Introduction

Extreme heat poses a significant and growing global threat to human health [1,2,3]. In Canada, heat is a leading cause of weather-related mortality, linked to hundreds of deaths and thousands of emergency department visits each year [4,5,6]. The threat posed by extreme heat events, including heatwaves and heat domes, is projected to increase in frequency and severity as a direct result of climate change [7,8]. Individual- and population-level exposure, vulnerability, and adaptive capacity to such events are shaped by socioeconomic and ecological conditions, as well as long-standing sociopolitical structures that reproduce health inequities [9,10,11]. The health impacts of extreme heat are thus disproportionately borne by certain populations, including older adults, socially isolated individuals, children, those with mental and physical health conditions, those of lower socioeconomic status, and those who experience precarious employment or housing [6]. As the health effects of extreme heat are localized, actions to mitigate the negative health impacts of extreme heat should be tailored to best respond to community needs [1,12].

Municipalities remain at the forefront of extreme heat preparedness and response activities that often span multiple sectors within and beyond government [9,13]. For example, features of the physical environment, such as green spaces, shade structures and public water fountains, coupled with housing standards and residential tenancy laws, may help mitigate negative heat health impacts among those at greatest risk due to social and material deprivation [9]. Moreover, occupational health and safety legislation can protect those working in extreme weather conditions [4,14], whereas public transit systems may be designed to reduce prolonged heat exposure and improve access to heat respite services (e.g., cooling centres) [15]. And while public education campaigns can improve heat health literacy and health promotion among the general public [16], regular engagement between government sectors and community-based organizations can further align government-led heat response strategies with the needs of those directly impacted [9,11]. Effectively and equitably mitigating the negative health impacts of extreme heat may therefore be strengthened through deliberate ‘intersectoral’ coordination—that is, the alignment of strategies and resources across government portfolios (i.e., ministries, divisions, or agencies) and between government and non-government sectors (e.g., community organizations) [9,17,18].

In addition to the above ‘horizontal’ collaboration across local sectors, addressing the intersecting health impacts of extreme heat also requires ‘vertical’ collaboration between levels of government [19,20]. Vertical collaboration is particularly important in federal systems [13,19], where different governments may have overlapping authority over public health, as well as the social and ecological determinants that give rise to differential vulnerability and exposure to heat [9].

While ideal, several challenges surround collaborative efforts. Local governments are limited by financial and human resources, and may experience negative service spillovers from implementing strategies and responding to extreme weather events, such as increased operational costs from expanding cooling centres [21]. Horizontal collaboration may also be hindered by a lack of dedicated leadership to address climate-related issues, siloing between sectors, or the low political salience of climate-related issues [22,23]. On the other hand, vertical collaboration may be constrained by high issue polarization and ideological tension between levels of government, which may limit the acceptability and feasibility of local action [22]. Collectively, these challenges may weaken the intersectoral ‘policy coherence’ necessary for effective heat response [24], leading instead to fragmented, duplicative, or maladaptive policies and strategies [9,13]. This may, in turn, increase the risk of what are ultimately maladaptive and inequitable extreme heat response strategies.

Efforts to effectively and equitably address the health impacts of extreme heat may therefore be strengthened by a robust understanding of effective governance mechanisms for collaborative climate action. Canada provides the ideal jurisdiction to explore these challenges and uncover possible strategies for strengthening governance mechanisms. Like other high-income countries, Canada is facing increasing frequency and severity of heatwaves, with inequitable and highly localized impacts [4,9,25]. Moreover, Canada is a highly decentralized federation wherein efforts to address the health and inequitable impacts of climate change span national, sub-national (provincial/territorial), regional, and local jurisdictions [13]. Within the public health system specifically, while provinces and territories are primarily responsible for administering public health programs and services, the role of local governments in public health activities varies across the country [26,27]. The federal government also plays a leadership role in some areas, along with some financing, technical advisory and convening roles [9]. Notably, Canada’s Chief Public Health Officer has identified intersectoral and collaborative action on climate change as a critical knowledge gap in both climate and public health governance [9]. To address this gap, our study sought to identify and assess governance mechanisms shaping local heat response efforts involving public health and other intersectoral stakeholders in Canada. To do so, the following research question was addressed:

*How and to what extent do existing governance mechanisms shape the nature and scope of collaborative extreme heat response across sectors in Vancouver, Toronto, and Montreal?*

For this study, governance was conceptualized as the formal and informal structures and mechanisms that condition the setting, pursuit, and measurement of progress toward common goals, and that establish roles and interactions among government sectors (e.g., ministries or divisions) and between government and non-government sectors (e.g., community organizations) [28,29].

## 2. Materials and Methods

### 2.1. Conceptual Frameworks

Our research was informed by theories of governance that involve collaboration across multiple sectors and scales to address complex, multi-faceted issues such as climate change [21,30,31]. Such complexity, for example, includes the jurisdiction-spanning externalities of climate policies across policy sectors at local, regional, and national scales [32]. Polycentric governance accounts for multiple ‘centres’ of semi-autonomous decision-making that together constitute a relatively interdependent and coherent system of relations [33,34]. While competition and conflict exist—for example, with regard to public policy development and the provision of public services [34]—each centre is said to adhere to an overarching set of rules or mechanisms to promote cooperation [33,34]. The capacity to self-organize is an ideal quality of polycentric arrangements, which, in turn, both shapes and is shaped by collaboration [35]. Within this framework, decision-making authorities can voluntarily undertake collective climate action while self-regulating collaborative efforts [21].

Moreover, multilevel governance accounts for the complexity of climate policymaking within federated countries like Canada [30], including horizontal and vertical interactions. Horizontal relations within government (e.g., between ministries) and between government and non-government actors (e.g., community organizations, advocacy groups) [34] may be needed to overcome institutional fragmentation and deliver on crosscutting policy issues [22,36]. Vertical governance relationships tend to be formally prescribed, with each level adhering to defined policy jurisdictions [30]. While the autonomy of local governments “nested” within higher-level (i.e., provincial, federal) institutional frameworks may be limited to some degree [22,37], it is generally agreed that neither ‘upper’ nor ‘lower’ levels can effectively fulfil their respective functions without vertical coordination [21,36]. These frameworks, therefore, help identify the roles of the diverse actors involved in climate policy and how relationships are managed in effective climate action [24,30].

Provan and Kenis developed an assessment framework for complex intersectoral networks [31]. They define a ‘goal-directed network’ as three or more autonomous organizations collaborating to achieve a collective outcome [31]. Such networks exist across both public and nonprofit sectors, where coordination is needed to effectively address complex policy issues [31], including polycentric and multilevel arrangements. Provan and Kenis describe three forms of network governance:

- *Participant-Governed Networks* are decentralized, with shared responsibility among member organizations in terms of managing internal roles and operations, and collective decision-making. Participants interact frequently and directly to achieve a common goal.
- *Lead Organization-Governed Networks* are centralized arrangements that rely on a single participant organization to facilitate network activities. Power among participants is asymmetrical, and members frequently interact to achieve network goals that may closely reflect the interests of the lead organization.
- *Network Administrative Organization* is a centralized arrangement relying on an external entity (individual or organization) to strategize network priorities and coordinate operations. The distribution of power is asymmetrical, with board structures used to convene infrequent interaction among member organizations.

According to Provan and Kenis, the likely effectiveness of these governance forms depends on several ‘critical contingencies’, including (1) level of trust; (2) number of participants (i.e., network density); (3) network goal consensus; and (4) network-level competencies [31].

To further assess the governance mechanisms supporting intersectoral coordination within network arrangements like those above, we drew on Greer and colleagues’ TAPIC framework [38]. The TAPIC framework consolidates mechanisms into five overarching domains of effective governance: (1) *transparency* of decision processes (supported by mechanisms such as regular reporting or public meetings); (2) *accountability* for all actions (e.g., codes of conduct or financial mechanisms); (3) *participation* among interest holders (e.g., advisory committees, public forums); (4) *integrity* of the work conducted (e.g., internal or external audits, legislative mandates); and (5) *capacity* of the policy bureaucracy (e.g., research capacities, staff training) [38].

Together, these frameworks informed the development of our semi-structured interview guides and guided the deductive analysis of interview and documentary data. They provide a structural lens to support our effort to identify, describe, and assess existing collaborative climate governance arrangements shaping extreme heat response in Vancouver, Toronto, and Montreal.

### 2.2. Data Collection

We conducted a comparative case study of the governance mechanisms that shape collaborative climate action in Canada’s three most populous provinces. Given the significant contribution of extreme heat to weather-related mortality in Canada, we selected the formal heat response plans of three cities—Vancouver (British Columbia—BC), Toronto (Ontario), and Montreal (Québec)—as comparative cases. Heat response within these major cities is nested within provincial arrangements, and features of their respective public health systems were anticipated to reveal a range of distinct yet comparable governance mechanisms at play.

Data were collected through key informant interviews and triangulated through documentary analysis of materials related to extreme heat response in each city [39,40]. Findings were synthesized descriptively and thematically to characterize governance arrangements shaping local heat response in each city, as well as to identify related strengths and challenges. This study received ethics approval from the University of Toronto Research Ethics Board (#45551).

#### 2.2.1. Document Review

Building on a rapid scoping review of extreme heat response in BC, Ontario, and Quebec [41], we purposively retrieved both publicly available and internally circulated documents from key informants relevant to each case between November 2023 and December 2024. Sources included peer-reviewed literature, government and technical reports, climate, public health, and emergency response legislation, government documents, strategic and operational plans and frameworks, news articles, executive memos, and meeting minutes. In total, 30 documents were included, spanning multiple levels of government and policy sectors. Internally circulated documents were provided by key informant interviewees. Documents were reviewed and synthesized into preliminary case overviews prior to conducting interviews, ensuring that interview guides were context-specific.

#### 2.2.2. Interviews

We conducted semi-structured interviews with key informants involved in local heat response within each city. Prospective key informants were identified through documentary analysis and case-specific experts (such as public health officials) within the broader research team. We also employed snowball sampling by asking key informants to connect us with other individuals involved in heat response within each province of interest.

Interviews were conducted by two research team members (M.S.S.S. & S.S.) between April and October 2024 using an interview guide piloted with an advisor with expertise in local extreme heat response, governance, and public health policy (Appendix A). For interviews with key informants from Montreal, the guide was translated into French by a bilingual team member (M.S.S.S.). Interviews were conducted virtually and video recorded via Zoom in English (n = 17) and French (n = 11), and lasted approximately 30–90 min. English interviews were transcribed using Otter.ai software (2024), and Zoom 6.0 transcripts were used for the French interviews. Transcripts were edited for accuracy by team members (S.S., M.S.S.S., and a research assistant). Recruitment ended when it was agreed by the research team that thematic saturation had been reached in each case [42].

### 2.3. Analysis

Our analysis sought to identify and characterize the governance mechanisms shaping collaborative extreme heat response within each case, and to gauge both the measured (e.g., through formal evaluations or reviews) and perceived effectiveness of said mechanisms. To do so, we conducted a thematic analysis of the case-specific heat response materials and interviews using NVivo 14 qualitative software. A preliminary codebook based on our interview guide, and informed by the aforementioned network governance and TAPIC frameworks [31,38], was developed to guide deductive coding (Appendix B), and inductive coding was used to capture new ideas. English interviews were coded by S.S. and a research assistant, and French interviews were coded by M.S.S.S. Up to three interviews were double-coded per case to ensure consistency between the two coders and discrepancies or challenges were resolved through consultations with the Principal Investigator (S.A.).

Using case-specific documents and interview data, we constructed organigraphs to illustrate and compare the collaborative networks of each case, including their levels of complexity and network integration. Organigraphs are useful for visually depicting the relationships between intersectoral actors, and identifying where collaborative and accountability mechanisms may be strengthened [43]. We employed member-checking by inviting study participants from each case to review our results, including the organigraphs. Five participants from Montreal and Vancouver provided written or verbal feedback to help clarify and contextualize our findings, and to confirm their accuracy.

## 3. Results

### 3.1. Key Informant Characteristics

Of the 42 individuals invited to participate, we interviewed a total of 28 key informants across the three cases, including past and present representatives from local and regional public health authorities, municipal government divisions, regional service organizations, and community organizations. Table 1 displays the participants by case and sector. Fourteen invited individuals did not respond or declined to participate due to a lack of involvement in or knowledge regarding local heat response, and there were no withdrawals.

### 3.2. Overview of Heat Responses

This section provides brief descriptions of the heat response plans and actors involved in each case. Details of their specific components can be found in Table 2 and the subsequent thematic analysis sections.

#### 3.2.1. Federal Role

Environment and Climate Change Canada (ECCC) is the key federal government actor contributing to local heat response in each case. In BC and Ontario, ECCC uses regional criteria to issue early notifications and heat warnings to health and emergency management sectors through standard weather communication systems. ECCC heat warnings activate the local heat response plans of Vancouver and Toronto [44,46,48]. In Quebec, ECCC’s heat warnings are only one factor that public health departments consider when deciding whether to activate local heat response plans.

#### 3.2.2. Vancouver

**Figure 1:**
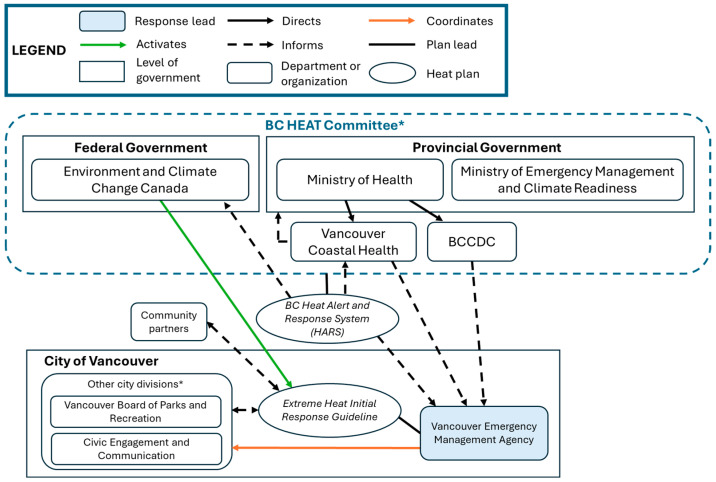
Organigraph of Vancouver’s heat response. * Not all actors are shown due to space limitations. BCCDC: British Columbia Center for Disease Control.

The City of Vancouver’s *Initial Response Guideline* outlines the response activities to be implemented during the early stages of a heat event [45]. The Vancouver Emergency Management Agency (VEMA), a division of the City of Vancouver, provides operational and strategic leadership of these activities before, during, and after a local Heat Warning (Level 1 activation) is issued by ECCC (Figure 1). The *Initial Response Guideline* draws on recommendations put forth by the BC Health Effects of Anomalous Temperatures Coordinating Committee (BC HEAT Committee), and follows the vertical heat alert activation system set out by the provincial Heat Alert and Response System (BC HARS) [50]. City-wide heat response activities include: (1) Cooling Centres activated within civic facilities and community organizations; (2) Outdoor Heat Respite, including shaded spaces, misting stations, and drinking fountains; (3) Community Outreach, including wellness checks and personal cool kit distribution; (4) Communication and Public Messaging of heat health information; and (5) Grants and Funding opportunities for community-based heat relief initiatives. In the rare event of an Extreme Heat Emergency (Level 2 activation), Vancouver’s emergency operations centre is automatically activated to support coordination among city departments and community organizations [44].

#### 3.2.3. Toronto

**Figure 2:**
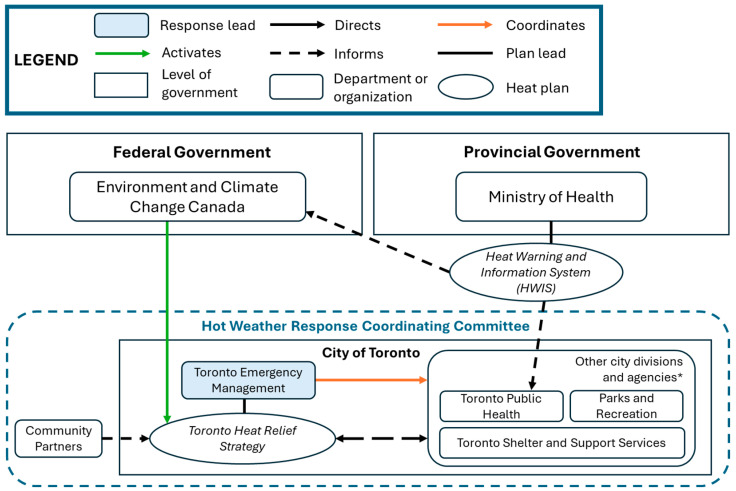
Organigraph of Toronto’s heat response. * Not all actors are shown due to space limitations.

Toronto’s *Heat Relief Strategy* outlines the protocol for local hot weather response between May and September of each year [46]. Toronto Emergency Management (TEM), a division of the city of Toronto, provides operational leadership of the Heat Relief Strategy, including the coordination of 18 municipal divisions and community partners comprising the Hot Weather Response Coordinating Committee (Figure 2). Members of the Committee administer heat relief services once a Heat Warning is issued by ECCC, consistent with the Ontario Harmonized Heat Warning and Information System (HWIS). These services include: (1) a Heat Relief Network of more than 600 public facilities providing respite throughout the summer; (2) public messaging, including heat health information and service availability; (3) cool spaces in apartment buildings measures to ensure residents of multi-unit housing remain informed of heat relief strategies; (4) street outreach for people experiencing homelessness; and (5) extended public hours of operation for public pools across seven city-wide locations [46].

#### 3.2.4. Montreal

**Figure 3:**
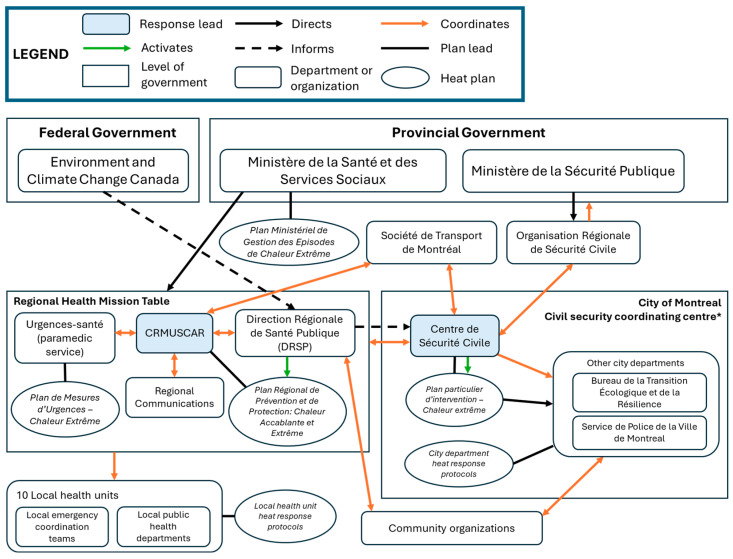
Organigraph of Montreal’s heat response. * Not all actors are shown due to space limitations. CRMUSCAR: Coordination régionale des mesures d’urgence, sécurité civile et accès réseau.

Montreal’s extreme heat response strategy is shaped by two core separate but aligned response plans: the city’s civil security centre (Centre de sécurité civile)’s *Plan particulier d’intervention—Chaleur extrême* and the regional health system emergency coordination unit (Coordination régionale des mesures d’urgence, sécurité civile et accès réseau—CRMUSCAR)’s *Plan Régional de Prévention et de Protection: Chaleur accablante et extrême* [47,48], developed in partnership with the regional public health department (Direction régionale de Santé Publique—DRSP). The CRMUSCAR and DRSP are designated as ‘regional’ as they provide leadership across all five health and social service centres (Centres intégrés universitaires de santé et de services sociaux—CIUSSS) covering the city of Montreal (though they are housed within one). In this way, the regional health system heat response plan covers the same territory as the municipal heat response plan. Both plans are updated annually. The city’s civil security centre, the CRMUSCAR, and the DRSP work closely together to ensure response alignment. The civil security centre convenes city divisions and regional health system actors such as the CRMUSCAR, the DRSP, and the paramedic service (Urgences-santé) to deploy their plan. Once the DRSP has determined that extreme heat criteria have been reached, the CRMUSCAR is responsible for coordinating the activation and deployment of local response plans within each of the ten health units (five CIUSSS and five independent hospitals) (Figure 3) [48,51,52]. Quebec’s Ministry of Health (Ministère de la Santé et des Services Sociaux) heat plan supports aligned heat responses across regions and coordinates support from provincial agencies [53].

The city’s heat response interventions include opening cooling centres and public water infrastructure, water bottle distribution to organizations serving unhoused populations, communicating heat health prevention information, door-to-door check-ins with high-risk populations, and special measures to protect city staff, such as firefighters, from heat [47]. The city’s Ecological Transition and Resilience Bureau (Bureau de la Transition Écologique et de la Résilience) also conducts heat illness prevention outreach to at-risk communities in the spring [49]. The health system heat response plan includes the following levels of activation: seasonal monitoring, alert, intervention, demobilization, and recovery and focuses on opening cooling centres and setting up transport to them, weather and health surveillance, and preparing health services to receive individuals impacted by heat [48].

### 3.3. Governance Mechanisms Shaping Local Heat Response

The following sections present governance mechanisms shaping the nature and scope of collaborative heat response in Vancouver, Toronto and Montreal, including provincial structures and frameworks, formal plans, and legislation.

#### 3.3.1. Provincial Heat Response Coordination Structures

Key informants generally reported little direct interaction with higher orders of government in the context of extreme heat. In each case, provincial governments provide broad guidance for heat response activities in an effort to strengthen the coordination and consistency of geographically defined heat alert systems (Table 3). BC and Ontario provide guidance on the operationalization of regional heat alerting systems, including the role of public health in supporting local response activities. BC and Quebec also utilize formal structures to convene multi-level heat response actors.

The *BC Heat Alert and Response System* (BC HARS) supports intersectoral preparedness and response to extreme heat events [50]. The system is premised on geographically defined weather thresholds according to which ECCC triggers a ‘Heat Warning’, thereby prompting the activation of local heat response plans [50]. Accordingly, Vancouver’s formal heat response plan, the *Initial Response Guideline*, is operationalized as part of a vertical ‘trigger and activation process’ [50]. The BC HARS is updated annually by the BC HEAT Committee, whose authority is derived from BC’s provincial *Public Health Act* (Table 3) [50]. As such, the committee is led by the BC Centre for Disease Control and Ministry of Health, and is inclusive of actors at provincial (e.g., BC Emergency Health Services), regional (e.g., health authorities) and local (e.g., the Union of BC Municipalities) levels [50]. The BC HEAT Committee provides recommended actions to guide intersectoral heat preparedness and interventions, including for public health authorities and local emergency management authorities. These recommendations are not prescriptive, but instead recognize the value of regional and local initiatives identifying and responding to community needs.

Ontario’s *Harmonized Heat Warning and Information System* (HWIS) closely resembles the BC HARS and aims to enhance the consistency of extreme heat response among local public health units (Table 3) [54]. Essential activities of the HWIS include notification and communication processes, with the provincial Ministry of Health offering supplementary guidance via the *Ontario Public Health Standards* on HWIS governance (roles, responsibilities, and decision-making structures), regional heat warning thresholds, and recommendations for proactive heat response activities, including local/community collaboration [54]. Like the BC HARS, Ontario’s HWIS is non-prescriptive, recognizing that local-level heat response varies according to heat health surveillance, vulnerability assessments, local climate, and community needs. Regional HWIS thresholds guide ECCC’s issuance of a ‘Heat Warning’ and directly activate Toronto’s *Heat Relief Strategy* [54].

Quebec’s provincial public health institute, Institut National de Santé Publique du Québec, hosts a weather surveillance and warning system, known as *SUPREME* (Système de surveillance et de prévention des impacts sanitaires des événements météorologiques extrêmes), which transmit real-time extreme weather alerts to health system and civil security departments [55]. The Ministry of Health’s Extreme Heat Management Plan aligns heat response mobilization and communications across regions and outlines the roles of various provincial and regional health and social services actors (Table 3) [53]. The plan is mobilized and actors are coordinated by the Ministry’s civil security department, Direction générale adjointe de la sécurité civile et des affaires institutionnelles, and the provincial public health department, Direction générale de la santé publique. In parallel, the Regional Organization for Civil Security for Montreal, Organisation Régionale de Securité Civile, operating under the Ministry of Public Security, convenes provincial ministries and agencies when emergency plans are activated, following the DRSP activation for extreme heat and according to the provincial civil security plan [56]. The Regional Organization for Civil Security communicates local public health recommendations to provincial services, such as the Ministry of Education and the Ministry of Family, which are responsible for daycares.

#### 3.3.2. Clear Articulation of Roles and Responsibilities

Key informants identified clearly articulated roles and responsibilities as an important facilitator of collaborative heat response. However, these vary in formality across sectors and cases. While emergency management divisions have clear mandates to lead extreme heat response in each city, leadership in Montreal is shared with the health system, notably the Regional Public Health Department. Moreover, while extreme heat is largely viewed as a public health issue across cases, the roles of public health authorities varied, with Montreal’s assuming the most ‘hands-on’ approach. Across cases, the roles of supporting ‘operational’ actors are clearly outlined within either public-facing or internal plans, thereby enhancing heat response preparedness and execution.

**Heat Response Leadership.** In each case, emergency management divisions assume full or partial leadership of local extreme heat response (Table 3). Legislated mandates support network leadership by assigning clear, sector-specific authority while also prescribing the scope of work among sectors involved. 

VEMA leads Vancouver’s heat response planning. Although a division of municipal government, two pieces of provincial legislation assign VEMA’s leadership role: whereas the *Public Health Act* requires local governments to address “health hazards” generally, the *Emergency and Disaster Management Act* requires that local authorities, or their designated emergency management organization, oversee jurisdiction-specific emergency management activities [57]. In the event of a Heat Warning, VEMA provides administrative leadership by coordinating the city and community partners that administer the heat response activities specified within the *Initial Response Guideline*. Where an Extreme Heat Emergency is triggered, VEMA staffs a community partner liaison to conduct intersectoral coordination and communication efforts. And finally, VEMA leads iterative strategic updates of the *Initial Response Guideline* through regular consultation with subject matter experts, and based on feedback obtained through post-event debriefs that occur after each Heat Warning is terminated. 

TEM is the city division mandated under municipal bylaw to support the City of Toronto in preparing and responding to emergency events [58]. Accordingly, TEM also oversees the administrative components of Toronto’s *Heat Relief Strategy* by coordinating the Hot Weather Response Coordinating Committee [46]. In doing so, TEM ultimately seeks to prevent escalation to a Heat Emergency and attendant strain on health and social service sectors. 

Heat response in Montreal is co-led by the city’s civil security centre and the health and social services system, with an influential role for the Regional Public Health Department (DRSP). The city’s civil security centre receives its mandate to manage public emergencies from Montreal’s civil security plan [59]. The Ministry of *Health’s Extreme Heat Management Plan*, implemented in 2019, directs health emergency coordination units and public health departments to coordinate heat responses [53]. However, Montreal’s development of a local heat response as early as 2004 was largely driven by the local public health department’s initiative and commitment to the prevention of negative health impacts and by leveraging their established expertise. The resulting co-leadership between the health and social services system (represented by CRMUSCAR and DRSP) and the city’s civil security centre includes aligning respective heat response plans and coordinating related interventions, decision-making, and communications within their jurisdictions (i.e., the municipal government and ten local health units). The relationship is formalized by the 2022–2030 climate action collaboration agreement between the DRSP and the city [60]. Key informants explained that effective co-leadership is in part enabled by closely aligned priorities being protecting population health and ensuring people’s security, respectively. Both also share the objective of ensuring the continuity of services [48,59].

**Public Health Authorities.** While key informants generally recognized extreme heat as a public health issue, the roles of public health authorities vary. In each case, public health supports public communication and contributes subject matter expertise regarding heat health impacts. Of the three cases, Montreal’s regional public health authority assumes the most direct role by activating heat responses based on internal analysis and working closely with the city’s civil security centre to coordinate population-level interventions. By contrast, the Vancouver Coastal Health’s (VCH) public health officials advise on the strategic direction of local heat response efforts, whereas Toronto Public Health’s (TPH) role is almost strictly administrative. In each case, the nature and scope of public health involvement in extreme heat response were generally considered appropriate, while some highlighted aspects to be strengthened.

In BC, VCH is the regional health authority responsible for the provision of population and preventative health programs and services to a geographically defined catchment area of the City of Vancouver. Per the provincial *Public Health Act (2008)*, VCH is required to provide leadership and support in preventing environmental health threats [61]. Accordingly, VCH public health officials support VEMA’s leadership by lending subject matter expertise regarding best practices, strategic direction, and near-real-time heat health data. At times, VCH also acts as an intermediary and advocate on behalf of local climate actors, including VEMA, by relaying ‘on-the-ground’ concerns and issues to key climate actors, including, for example, the provincial Ministry of Emergency Management and Climate Readiness and Ministry of Health, and at the federal level, ECCC. While VCH’s roles reflect several recommended actions for public health actors put forth by the BC HEAT Committee [50], informants indicated that their contributions are largely voluntary, based on existing resources and capacities rather than a formal mandate or action plan.

[The heat response plan] isn’t the driver of our collaboration [with VEMA]. It’s more due the fact that they have specific capacities, and we have specific capacities […] so, in that way, the plan isn’t super important. We don’t have a specific role that we have to respond to. We’re not in that plan specifically […] We view [VEMA] as driving [heat response] and us supporting in whatever way we can.

(Vancouver Participant #7, Regional Health perspective)

Operating within Toronto’s municipal government, TPH contributes to local heat response according to several broad requirements outlined within *the Health Hazards Response Protocol* (e.g., communications) [62] and *Healthy Environment Climate Change Guidelines* (e.g., promoting healthy built environments) [63] of the *Ontario Public Health Standards* [64]. As the Ontario Public Health Standards specify minimum requirements for the provision of local public health services and programs, TPH’s roles and responsibilities are flexible rather than prescriptive.

I think [the *Ontario Public Health Standards*] do give a lot of flexibility to public health units to determine how they want to meet the [climate-related] requirements […] It’s kind of like, how do you interpret it for your own community? I think they’re intentionally left a bit vague for that purpose.

(Toronto Participant #6, City perspective)

The flexibility of this approach is exemplified by TPH’s departure from its original extreme heat leadership position in 2018. In this regard, key informants agreed that Toronto Emergency Management was better suited to oversee the *Heat Relief Strategy*, given its predominantly administrative nature. TPH’s primary responsibilities have since shifted toward maintaining the Heat Relief Network (i.e., cooling stations) and acting as a heat health ‘educator’ through public communications. This narrower set of responsibilities more closely reflects the stated requirements of the harmonized *Heat Warning and Information System* (HWIS), including communication of health protective measures, local partnership, and planning activities [54]. 

In Montreal, extreme heat is recognized as primarily a public health issue by government authorities, and as a special case among extreme weather emergencies. As a result, public health departments, particularly the DRSP, serve as subject-matter experts and assume both coordinative and advisory roles. Their responsibilities include heat surveillance, defining heat plan activation criteria, activating the heat response (through CRMUSCAR), and ensuring that it remains evidence informed. They achieve this through reviewing the heat strategy on a yearly basis, consulting emerging research evidence and heat mortality data collected by the paramedic service.

Since it’s a health issue, there are two elements: One, the entity that says whether it’s dangerous or not is public health. […] The criteria are defined by them, and then when it’s hot, that’s where it’s the CIUSSS that will be capable of really following what’s happening with public health and who will guide our operations to see where we need to intervene, where there’s a gap in capacity, where they need help.

(Montreal Participant #4, City perspective)

The DRSP’s influential role in intersectoral action is unique among the three cases, as it works closely with the city’s civil security centre to coordinate population-level interventions. Local public health departments also provide heat illness prevention resources to services and community groups involved in heat response through pre-established networks, to be communicated with the public and to target populations, as well as conducting media interviews. 

**Operational actors.** The roles and responsibilities of actors supporting the administration and operationalization of extreme heat response activities largely reflect their sector-specific mandates and scopes, which are more prescriptive than those of the public health sector. In each case, their roles and responsibilities are articulated within respective heat response plans. Leveraging existing mandates was perceived to strengthen the intersectoral coherence and effectiveness of multifaceted heat response efforts.

Vancouver’s internal *Initial Response Guideline* outlines the roles and responsibilities of city departments and partnering agencies leading up to and during Heat Warnings and Extreme Heat Emergencies [45]. These roles and responsibilities largely build upon and adapt the expert recommendations within the BC HARS guidelines while also leveraging existing departmental and organizational mandates. For example, as the BC HARS recommends that municipal authorities “undertake community outreach focusing on susceptible and high-risk populations” [50], key informants from the City of Vancouver explained that they deliberately engage organizations with existing resources and community rapport to maximize the impact of heat response efforts:

We have very intentionally selected organizations that function as hubs in the community and are already doing intersectional work. So, they already have networks that can fan out a little bit, and we focus on keeping it small and tight based on group that are already driving [local heat response] work.

(Vancouver Participant #1, City perspective)

Similarly, Toronto’s *Heat Relief Strategy* outlines the roles and responsibilities of the 18 city divisions, agencies, corporations, and community partners that comprise the Hot Weather Response Coordinating Committee [46]. In the event of a Heat Warning, the committee coordinates and administers response activities aligned with their existing mandates and operational capacities.

Montreal’s city civil security plan sets out “Missions” that mandate city department roles in emergency response and provide mission-specific coordinating tables [47,59]. For example, the “Order and Peace” mission gives the Montreal Police Service a role in door-to-door outreach during an extreme heat emergency. The city’s civil security centre invites all city-level missions to the first emergency coordinating centre meeting, which elect to participate if impacted. The province also outlines mission mandates in the provincial civil security plan, in which the Health Mission gives a mandate to all health region establishments in emergencies [56]. The CRMUSCAR convenes “Health Mission” actors at a regional-level coordinating centre (Figure 3). Specific services involved in heat response have protocols outlining more detailed roles for the layers of their organization, aligned with the CRMUSCAR and Montreal Civil Security lead plans.

#### 3.3.3. Structures Facilitating Network Collaboration

Formal structures are in place to facilitate intersectoral collaboration and communication in each case. Annual pre- and post-season meetings provide opportunities for actors to review roles and responsibilities, update response activities and services, and raise related concerns. Beyond these bi-annual meetings, there is notable variability in the frequency of collaboration and communication across cases, as well as the scope of actors involved (Table 4). Despite the presence of formal collaborative structures in each city, the portfolio-specific contributions of municipal actors in Toronto and Vancouver were perceived by some informants as ‘siloed’. In Montreal, response efforts were generally described as integrated, with plans aligned across city and health actors, and participants more aware of one another’s roles and responsibilities. 

Collaboration in support of Vancouver’s *Initial Response Guideline* occurs regularly among a broad scope of intersectoral actors. These collaborations are facilitated through three types of ‘coordinating calls’ convened by VEMA. The first are pre-season readiness calls with city divisions, community partners, and regional (e.g., VCH) and provincial (e.g., BC Housing) authorities. At times, these extend to neighbouring jurisdictions to compare and amplify heat response initiatives and avoid the duplication of efforts. Next, communication throughout the heat season largely coincides with Vancouver’s two-tiered heat alert system, with calls held to prepare for activation of Heat Warnings and Extreme Heat Emergencies. And finally, post-event debriefs occur after each lower-level Heat Warning is terminated. As such, key informants noted that debriefs occur regularly during heat season, with feedback informing real-time adjustments to the *Initial Response Guideline*. Despite the relative frequency of intersectoral coordinating calls among local and regional actors, key informants generally agreed that the nature of heat response efforts remains siloed.

We don’t necessarily know what engineering has planned, for example. But we don’t need to know, because we don’t directly interact with them. We go through VEMA, and they coordinate our responses.

(Vancouver participant #5, City perspective)

By contrast, formal interaction among members of Toronto’s Hot Weather Response Coordinating Committee is largely limited to pre- and post-season meetings during which roles and responsibilities are established, challenges or concerns voiced, and recommendations put forth. Committee meetings are overseen by TEM, which also acts as a point of contact to coordinate intersectoral heat response efforts throughout the heat season. Involved sectors are typically represented by a delegate who relays information to and from Committee meetings. While key informants generally reported heat response planning and execution to be supportive and collaborative, they also indicated little awareness of the nature and scope of sector-specific operations beyond their own.

I think at a high level we’re aware of what each other are doing, because we come to the table together and figure out how we can each contribute. And everything is documented. But we’re not privy to, say, how public health makes decisions, and engages with other sectors and shares information.

(Toronto participant #4, City perspective)

In Montreal, the heat response is coordinated through the city’s “Emergency Measures Coordination Centre”, which is a formal hierarchical structure with four levels of coordination (political, strategic, tactical, and operational), with the civil security director acting as the final strategic decision-maker [59]. However, the civil security division takes a “concertation” (i.e., cooperation) approach to coordination and decision-making during emergencies, including extreme heat. This approach involves convening all relevant city departments and external agencies (CRMUSCAR, DRSP, and Société de Transport de Montreal) to plan the response based on the existing formal strategy, including addressing needs across sectors and mobilizing resources in real-time, as well as collective problem-solving.

The way we work is that when we declare an emergency, we call [all the services], we describe the problem, we announce that we’re initiating, and we ask people if there’s a relevant element for them or not. […] And we’re really interdisciplinary, so [we] could have 34 people sitting, with 34 different expertise. [We] can’t possibly know all of the potential impacts on the 34 [services] and [we] can’t know their detailed operations… People will often bring up something you would have never thought of. So it works really well honestly.

(Montreal participant #4, City perspective)

Communications during extreme heat activation pass through the city coordinating centre, increasing clarity and transparency across services and with the public. Montreal’s health system emergency coordination unit (CRMUSCAR) coordinates heat response across the ten local health units through the “Health Mission” table, which includes representatives from public health, healthcare system, and Urgences-santé (paramedic service). Both coordinating centres conduct pre-season meetings with their respective services to ensure response preparedness, and host coordinating meetings and communications throughout active responses. CRMUSCAR, DRSP, and the city’s civil security centre work to ensure alignment between their responses and take a horizontal approach to coordinating actors. 

Quebec is also the only province with formal structures at the regional level (i.e., beyond City of Montreal geographic boundaries). While the Regional Civil Security Organizations coordinate with provincial agencies (as described above), they also play an advisory role to municipalities in managing emergencies. They facilitate vertical communications between local/regional agencies and the provincial government, as well as with neighbouring regions, to ensure harmonized heat alerts. 

#### 3.3.4. Leveraging Informal Networks

Key informants across cases reported engaging with informal networks as a useful strategy for strengthening intersectoral collaboration and advancing heat response initiatives. In Vancouver, key informants described “floating ideas” and making “cold calls” to contacts across sectors in an effort to troubleshoot issues, and considered ‘mundane’ conversations between colleagues to be useful for voicing complaints and working through problems.

There’s an interplay that often happens informally. It’s not gossip, it’s chatting about issues and airing what is and isn’t working mutually, and then taking that back and floating it to the right people.

(Vancouver Participant #8, Regional Health perspective)

These approaches were noted to contrast formal processes used to address more substantive and public-facing issues requiring official medical or public health support for a heat response initiative.

Key informants in Toronto described similar strategies, with particular focus on leveraging more ‘informal’ conversations to identify strengths and ongoing challenges of specific initiatives. The development and implementation of Ontario’s HWIS was highlighted by a key informant as a notable example of how everyday conversations can lead to substantive change:

Over time we began to work with the same group of players on heat. My colleagues and other health units had conversations about [the inconsistency of cross-region heat alerts]. So it was through those conversations that we identified it as a pressing issue to our colleagues at Health Canada and Environment Canada. And as a result, we worked together over the next five years to develop a harmonized heat warning system.

(Toronto Participant #2, public health perspective)

In Montreal, informal networks were said to develop over time through various initiatives such as pilot and research projects. For example, the Resilient Hub pilot brought together and funded new local government and community organization collaborations. Informants emphasized the importance of maintaining regular communication beyond times of crisis to build rapport and trust, and preserve long-term relationships as individuals change positions and organizations.

The quality of the collaboration isn’t worked out during a heatwave. It’s worked on in advance, and is crystallized over several months or years. It’s in this way that you’re able to arrive and be effective in collaboration. If you take all new actors, you put them in a room, you wouldn’t have the same level of quality in the response. In my opinion, it’s the experience in emergency response that strengthens the team, it’s the quality of the network, the reinforcement of collaborations prior to events occurring.

(Montreal Participant #9, police perspective)

Creating networks with a common goal or priority, such as the “Réseau Résilience Ainé.es Montréal”, can provide a structure for relationship-building and communications.

### 3.4. Areas for Improvement

Community-engaged heat response planning and implementation, strategic evaluation, and accountability constitute key areas for improvement within each case. Commonly cited barriers to these activities include budgetary constraints, limited trust and capacity (‘know-how’), and difficulty in identifying and measuring the impacts of public health interventions. Together, these limitations pose notable implications for how existing heat response efforts effectively address equity concerns.

#### 3.4.1. Strengthening Heat Health Equity Through Community Engagement

The heat response plans of each city clearly acknowledge the disproportionate health impacts of extreme heat experienced by certain populations, including older adults and young children, people with chronic illness, and socially disadvantaged communities [44,46,48]. Although ‘health equity’ is not explicitly referenced within the plans, key informants highlighted efforts to enhance equitable heat response. For example, in addition to targeted outreach to high-risk populations (see Table 2), the City of Vancouver’s public-facing website offers heat health information resources in 12 languages [65]. Similar strategies are employed by the City of Toronto, which, since 2019, has additionally expanded its ‘heat relief network’ to more than 600 city-wide facilities to enhance the accessibility of cooling centres among diverse populations impacted by extreme heat. In Montreal, key informants described conducting data analyses to identify ‘at-risk’ populations by neighbourhood, which then inform response activities such as targeted heat health communications and door-to-door check-ins during heat emergencies (Table 2).

Key informants across cases and sectors generally recognized the value of community-engaged heat response planning, providing notable examples of such activities. However, several challenges limit their nature and scope. For example, following the 2021 heat dome, substantive updates to Vancouver’s *Initial Response Guideline* included strategies to leverage community capacities to support local extreme heat response [44]. In 2022, Vancouver received funding to pilot several community-based initiatives, including volunteer heat response training and education, and expanding public transportation for at-risk populations [44]. Key informants identified the benefits of these initiatives to include leveraging existing trust and rapport between community organizations and populations experiencing stigma and marginalization, which, in turn, improved the city’s ability to identify and respond to community needs. At the same time, however, informants expressed frustration regarding the complex and stringent nature of the funding criteria attached to these initiatives, which ultimately limit the services, community organizations, and timeframes the city can support.

A lot of community organizations have close ties with vulnerable communities. They’re trusted and familiar. But they’re not getting funded for any heat response because of how strict the criteria are. So, trying to find a way for that to happen would help us reach those who are most at risk.

(Vancouver Participant #8, Regional Health perspective)

Beyond funding initiatives, VEMA regularly partners with VCH to host community roundtables to ensure that the needs of various groups are identified and addressed. These efforts are supplemented through additional outreach activities, including interviews, focus groups, and surveys, to better understand community needs.

As much as possible, our work is being informed and driven by community needs. We’re informed by the right groups rather than making assumptions about what is needed. We find out what is needed pretty immediately, and respond the best we can.

(Vancouver Participant #1, City perspective)

By contrast, community engagement in Toronto is limited to the few community partners listed within the *Heat Relief Strategy*. Moreover, the topic of community engagement was rarely raised by key informants, save for some mention of past efforts to inform heat response through public opinion and telephone surveys. More recently, the Environmental, Climate and Forestry Division of the City of Toronto was noted to have engaged with community organizations as part of heat response initiatives beyond the formal *Heat Relief Strategy*. Generally, however, key informants agreed that sustained and meaningful community-engaged heat response planning and execution has yet to be achieved and, as such, constitutes an important area for improvement.

How do we work with community? How do they provide input into heat planning? How do we know the experiences of those living in these conditions? There’s a whole community engagement piece, and there is some work happening on this, but we don’t have a good systematic connection, and I’m not sure how effectively they’ve been engaged.

(Toronto Participant #1, City perspective)

Key informants consistently identified persistent barriers to community-engaged heat response planning, including limited funding to support community organizations, frequent turnover of municipal government actors, and a lack of capacity (time, ‘know-how’) among said actors to bring the full range of relevant community voices to the table in a sustained and meaningful way.

Although local heat response plans do not define a formal role for community organizations in Montreal, key informants indicated that they play an important role in spreading heat prevention awareness among underserved populations, particularly through sharing DRSP materials. The city’s Ecological Transition and Resilience Bureau, in particular, partners with and provides training for community organizations as part of their heat prevention outreach activities, as previously financed by the 100 Resilient Cities initiative [66]. Some community organizations were also noted to conduct their own check-ins on vulnerable community members during heatwaves. Despite these efforts, community organization representatives reported a disconnect between community needs and government response. They also highlighted a reduction in government financing over time, which limits community capacity to engage in extreme heat outreach and response.

The number one challenge is that the community is not heard. We bring up issues, that this isn’t working, or that your project doesn’t apply to our clientele, but we’re not heard. It’s too bad because it widens the gap between the DRSP and the Community sector.

(Montreal Participant #3, Community perspective)

#### 3.4.2. Strategic Evaluation of Heat Response Plans

When questioned, key informants across cases reported that they do not typically conduct formal evaluations of their heat response plans to ensure that they are effectively and equitably meeting their intended outcomes. In this regard, those from Vancouver and Toronto noted that, because their respective plans leverage existing facilities (e.g., recreation centres or public libraries as cooling centres) and outdoor locations (e.g., misting stations in public parks), it is difficult to monitor the extent to which these are used for the explicit purpose of heat respite.

I think there’s value in these metrics. But the question is around efficiency and effectiveness. It’s difficult, because these are public spaces. People go when they need to. So, we wouldn’t just say, while you’re here, can you check off a box to say why you’re visiting? Is it for heat? We can’t really request and effectively track that information from the public.

(Toronto Participant #3, City perspective)

Key informants from Vancouver echoed this challenge while noting that this issue is further compounded by the general difficulty in demonstrating the impact of preventative strategies.

It’s notoriously hard to evaluate the impact of public health programs, because success is when nothing happens, right? If nobody dies during the next heat dome, that’s a major success. But how do you prove that it was because of your interventions?

(Vancouver Participant #7, Regional Health perspective)

In addition to the lack of capacity needed to develop robust evaluation plans, key informants in Toronto cited additional challenges, which included time constraints imposed by relatively short heat seasons (i.e., May to September) and the rapid transition to focus on “cold season” preparedness. Although efforts to evaluate specific interventions, such as Cool Kit distribution, are currently underway in Montreal, and there is greater heat-related health data sharing between agencies such as Urgences-santé and DRSP, key informants expressed similar difficulties with evaluations more generally.

In the absence of formal outcome evaluations, key informants across cases reported ‘self-monitoring’ and ‘self-correcting’ their efforts to ensure their heat response activities are implemented as intended. In this regard, post-season debrief meetings were considered a key accountability mechanism through which key informants regularly report back on their activities and establish roles and responsibilities for the subsequent heat season. Although not ideal, key informants generally considered this to be a pragmatic and sufficient approach given the multitude of challenges shaping extreme heat response.

## 4. Discussion

Our comparative case study identified and assessed the effectiveness of governance mechanisms supporting intersectoral extreme heat response in the Canadian cities of Vancouver, Toronto, and Montreal. In each case, formal heat response strategies involve alert systems, the provision of heat health information, cooling centres and services, and outreach to high-risk populations. As such, extreme heat response largely entails horizontal collaboration, implicating multiple sectors within and beyond the realm of local government. Vertical network collaboration exists to a comparatively lesser extent across the provinces, with key informants from Toronto reporting no direct engagement with higher orders of government. However, local actors in Vancouver reported frequent engagement with VCH, which, as a regional health authority, also brokers local information to provincial and federal levels. Heat response in Montreal entails greater vertical collaboration, with regular engagement taking place between local, regional, and provincial actors. Federal involvement is notably limited in each case, with ECCC playing a central role in the activation of local heat response plans in Vancouver and Toronto and, to a lesser extent, Montreal. Together, these collaborative networks reflect both polycentric and multi-level governance arrangements.

### 4.1. Network as a Form of Collaborative Governance

Our findings indicate that the nature and scope of collaborative heat response vary across cases, which may be partially attributable to the form of network governance utilized [31]. To illustrate, both Vancouver and Toronto have adopted a ‘*lead organization-governed*’ network approach, whereby VEMA provides clear administrative leadership of the *Initial Response Guideline*, and TEM of the *Heat Relief Strategy*. The lead organization approach is said to be effective where large networks reduce the need and feasibility of direct engagement among member organizations, who instead rely on brokered network interactions [31]. This ‘critical contingency’ reflects the coordinative functions of both VEMA and TEM, and may partially account for the ‘siloed’ heat response efforts described by key informants. Siloing may be further embedded by the portfolio-specific contributions of each sector, perceived by informants to leverage existing mandates and capacities, and strengthen the intersectoral coherence of multifaceted heat response activities. In this regard, the network coordinative function of lead organizations ensures that the competencies of member organizations effectively support collective goal attainment [31].

Conversely, Montreal has adopted a more *‘participant-governed’* network approach, whereby the city’s civil security centre and the health system emergency measures coordination (CRMUSCAR) co-lead local heat response through alignment of their plans. Interestingly, while the effectiveness of the participant-governed approach is said to be contingent on a small number of participant organizations [31], the size and complexity of Montreal’s heat response network far exceed that of Toronto and, to a lesser extent, Vancouver. Montreal’s network seems to overcome this challenge by operating through multiple coordinating centres at municipal and regional levels, where coordination responsibilities are shared among different actors. Moreover, heat response efforts in Montreal were generally described as ‘integrated’, with key informants better attuned to the roles and responsibilities of various sectors than those in the other cases. This may reflect the relatively more decentralized nature of participant-governed networks, wherein members typically interact on an equal basis and contribute to collective decision making [31]. In Montreal, this is effectively illustrated by the ‘concertation’ (i.e., ‘cooperation’) approach used during extreme heat emergencies, though risk of decision-making paralysis is avoided by assigning final decision-making power to emergency management actors. Moreover, in notable contrast to Toronto’s *‘lead organization’* approach in particular, the integrated heat response within Montreal’s relatively decentralized network appears to support intersectoral coordination to achieve network-level goals [31].

### 4.2. Collaborative Governance Mechanisms Within Local Contexts

While network governance shapes the nature of intersectoral collaboration to some degree, our study also identified several consistent governance mechanisms across cases. This, in turn, calls into question why collaborative heat response may vary, despite similar mechanisms at play. In each case, network collaboration is goal-directed in that member organizations are deliberately convened to operationalize their respective heat response plans. Accordingly, goal-directed networks are said to evolve through conscious multilateral coordination within certain parameters [31], including existing policy and governance structures, and according to different mandates, institutions (norms, values) and resources available [34,67]. In the context of extreme heat resilience, collaborative configurations would also ideally reflect the health and social needs of impacted communities. Our case comparisons thus illustrate how similar mechanisms shape collaborative heat response activities, including their strengths and relative shortcomings, as follows.

The designated leadership of each local heat response plan is mandated at the municipal and provincial levels. However, only in Montreal are public health authorities explicitly called upon to respond to extreme heat, as articulated within the Ministry of Health and Social Services’ provincial *Plan Ministériel de Gestion des Episodes de Chaleur Extrême.* This gives Montreal’s response the added benefit of a more strategic, population health-oriented lens in managing extreme heat. This contrasts VEMA’s more general mandate for local “health hazard” response within BC’s *Public Health Act* and, to a greater extent, TEM’s municipal mandate to prevent emergency escalation. The relatively narrow focus of these latter mandates reflects both the administrative nature of VEMA and TEM’s leadership and, further, what is often the ad hoc nature of extreme heat response [68,69]. Nevertheless, each mandate appears to strengthen collaborative efforts by assigning clear authority among a multitude of heat response actors, while also prescribing the nature and scope of ‘heat response’ activities. This, in turn, enhances the integrity of local heat response in each case [38].

The integrity of collaborative heat response is further enhanced through the formal plans of each city, wherein the roles and responsibilities of network members are clearly articulated [38]. As earlier discussed, this approach ensures that, to the extent possible, each sector can effectively support the collective goal of local heat response. The public-facing plans of Toronto and Montreal’s health system, in particular, strengthen the accountability of heat response actors with regard to, for example, underperformance or misaligned priorities [38]. This may be especially critical where more formal accountability mechanisms are weak or lacking, as was reported by key informants. Although city councils technically oversee heat response efforts in these cases, informants generally reported ‘self-monitoring’ and ‘self-correcting’ their activities throughout heat season to ensure their responsibilities are fulfilled.

The heat response plans of each city additionally specify which sectors are formally ‘at the table’ with regard to heat response planning and implementation. However, there is notable variability in both the scope and frequency of stakeholder engagement across cases. In Toronto, participation is limited to the local actors comprising the Hot Weather Response Coordinating Committee, who convene bi-annually through pre- and post-season planning and debrief meetings overseen by TEM. Conversely, VEMA’s ‘coordinating calls’ typically involve both local and regional actors, including VCH. By contrast, Montreal’s heat response coordinating structures span the municipal civil security coordinating centre (inclusive of all relevant city departments), regional Health Mission Tables, other non-government regional agencies (Société de Transport de Montreal), and the Regional Organization for Civil Security, which liaises with provincial entities. Co-leadership across sectors in Montreal thus seems to enable greater intersectoral engagement and integration of heat strategies. In addition to pre- and post-season meetings, intersectoral engagement in Montreal and Vancouver occurs regularly throughout extreme heat season such that real-time adjustments are made to their respective heat response activities.

Findings from these case studies suggest that several governance mechanisms can facilitate more effective collaborative governance for extreme heat and other climate-related emergency responses. Local heat response actors can publish formal, public-facing heat response plans that articulate the roles and responsibilities of involved strategic and operational sectors, thereby strengthening response transparency, integrity, and accountability. Assigning specific roles should leverage existing mandates and capacities to strengthen the policy coherence of formal heat response plans. Heat plans should prescribe the nature and scope of heat response activities, and assign leadership among network actors. Where possible, leadership can be assigned according to the desired nature and scope of heat response efforts (e.g., assigning public health authorities as strategic co-leads may strengthen a population health approach). Heat plans may also outline intersectoral coordination structures to facilitate regular communication at all stages and levels of operation to improve clarity and transparency of actions during heat responses, and to support collective problem-solving.

### 4.3. Areas for Improvement: Community Engagement, Evaluation, and Equitable Heat Response

While our findings highlight some deliberate efforts to engage heat-impacted communities in response planning and implementation, key informants across cases considered this to be an ongoing area for improvement. Key barriers cited by community and government actors included funding and budgetary constraints, as well as limited trust and ‘know-how’. While these do not constitute issues of governance per se [38], they limit the scope of participation needed to achieve equitable resilience through effective and collaborative heat strategies [67,69]. This issue is further compounded by the absence of formal strategic evaluation in each city, underscored by the challenges in effectively measuring the impacts of heat response activities. While this challenge is not unique to our cases [70,71], actors rely instead on ‘self-monitoring’ and post-season debrief calls, which necessarily emphasize the processes (i.e., whether an activity was implemented as intended) rather than outcomes of response efforts (i.e., the plan’s efficacy in reducing negative heat health impacts) [38,72]. We therefore encourage future research to focus on community-partnered strategies and to effectively design and implement strategic evaluation of local heat response efforts. Heat response actors could ensure policy capacity among leadership, in particular public health actors, to enable the design and conduct of robust process and outcome evaluation both during and after the heat season. Doing so may allow heat response plans to be iteratively refined as needed, and may better support identification and response to the unique needs of diverse communities. Such efforts can enhance heat health equity and, in turn, strengthen accountability to community partners.

### 4.4. Implications for the Role of Public Health in Intersectoral Climate Action

And finally, we draw attention to the variable role of public health authorities in supporting extreme heat response in each case. The core components of each plan, including alerting systems, the provision of heat health information, cooling centres, and street outreach, have clear potential to mitigate the negative health impacts of extreme heat. As such, these ‘direct public health actions’ [9] reflect the typical nature of heat response in Canada and elsewhere [20,67] with preparedness and response plans, including early heat warnings, deemed a promising practice within ‘highly health-adaptive’ cities [73]. Per the *Ontario Public Health Standards*, TPH assumes a largely administrative role by coordinating city-wide cooling centres (the “Heat Relief Network”) while also supporting public communications regarding heat safety. In contrast, although not directly implicated within the *Initial Response Guideline* or corresponding public health legislation, VCH advises on the implementation and iterative refinement of local response activities by providing subject matter expertise and near real-time data upon VEMA’s request, including during regular heat event debriefs throughout extreme heat season. In Montreal, the DRSP assumes both coordinative and advisory roles through the provision of subject matter expertise, heat surveillance, and public communication and resources. Together, these cases effectively illustrate the potential spectrum of public health’s role in local heat response, with Montreal adopting the most comprehensive approach in terms of the core public health functions fulfilled [9].

While useful, the ‘direct public health actions’ of Vancouver, Toronto and Montreal’s heat response plans are largely situated downstream and, as such, function to address direct community- and individual-level risk to extreme heat [9]. This reflects the health orientation of several response efforts identified elsewhere [73], and may be partially attributed to focusing events such as the 2021 heat dome in Western Canada [4], which often lead to more ‘visible’ public health solutions to appease public concerns [71]. However, the downstream impacts of extreme heat are likely to worsen without greater attention to the structural determinants of extreme heat vulnerability [9]. Public health authorities may therefore consider how their leadership and expertise may support future efforts to systematically apply an equity lens to heat-related activities, including through the full range of public health functions [9]. Within our cases of interest, this will necessitate improved policy capacity to meaningfully engage community partners [9,74] and conduct robust strategic outcome evaluations through which heat response activities are iteratively refined [72]. Where the financial and human resources needed to support such efforts are limited, heat response actors may draw on existing strategies such as informal networking and interjurisdictional consultation to facilitate policy transfer and learning [67]. As opportunities arise, they may also seek to secure adequate funding or budgetary allocation, both internally within city divisions and externally to communities, to enable the scope of intersectoral engagement necessary for effective and equitable activities addressing extreme heat. And finally, the development of policy and legislative interventions may be strengthened through collaboration between public health and other sectors aligned with key social determinants of heat health inequity, including housing, social services, labour, health care, and infrastructure [9]. Together, these efforts are critical to resituating current heat response efforts further upstream and strengthening long-term community resilience to increasingly frequent and severe extreme heat events [75].

### 4.5. Limitations

Our findings may be limited by our focus on extreme heat response rather than the broader spectrum of local climate preparedness, recovery, and resilience efforts that may centre community voices and health equity goals. However, our analysis has uncovered community engagement as a key limitation of formal heat responses, which is critical for mitigating the negative health impacts of extreme heat events. Moreover, our analysis was unable to discern the directionality of existing governance arrangements — that is, whether network configurations have stemmed from the aims and objectives of case-specific heat response plans, or vice versa. Similarly, our analysis does not account for the different historical contexts and risks within our cases, and how these may shape the nature of existing heat response efforts. Our focus on large Canadian cities with well-developed public health systems may additionally limit the transferability of our findings to smaller jurisdictions with different public health and governance systems. However, local actors may usefully draw on our analysis of effective governance mechanisms to inform heat response efforts within their own contexts. In this regard, future analyses may be strengthened by the inclusion of a broader scope of potential heat response actors, including those from the healthcare sector and emergency responders, particularly in Vancouver and Toronto. As well, insights into the impacts of structural mechanisms, such as political ideology and power, may strengthen the development of strategies for upstream climate action. Finally, while both the case study methodology and our relatively small sample sizes may limit the generalizability of our findings, saturation was achieved in each case, and interview data were triangulated with a range of relevant sources.

## 5. Conclusions

This comparative case study provides an in-depth exploration of the collaborative governance mechanisms shaping local extreme heat response among intersectoral stakeholders in Vancouver, Toronto, and Montreal, Canada. Cities remain at the forefront of extreme heat response efforts — the inherently intersectoral nature of which requires both horizontal and vertical collaboration. And while public health has been deemed integral to leading collaborative climate action, our analysis illustrates that the roles and responsibilities of this sector vary, sometimes substantively, leaving room to realize the leadership potential so envisioned. Moving forward, it is critical that the health and social impacts of increasingly frequent and severe climate emergencies, including extreme heat events, are mitigated through upstream policy interventions to address the social and structural determinants of heat health inequity. Such efforts will ideally occur alongside future research seeking to strengthen climate actor capacity to meaningfully engage communities in heat response planning, as well as to conduct robust and systematic outcome evaluations of response activities. Effective and equitable public health and climate actions in this vein are necessarily context-specific. Together, these priorities are critical to effective intersectoral collaboration to equitably address extreme heat impacts within Canada and beyond.

## Figures and Tables

**Table 1:** Participants by case and sector.

	Montreal	Toronto	Vancouver
Total no. of participants	12	8	8
	**Representation by sector**		
Public health	✓	✓	✓
Emergency management	✓	✓	✓
Community organization	✓	✓	
Police/fire/paramedicine	✓		✓
Environment	✓		
City management		✓	

✓—indicates the sector of individual participants within each case. Note: The expertise of some participants spanned multiple sectors. Participant numbers are omitted to preserve anonymity.

**Table 2:** Extreme heat response plans by case.

Response Function	Vancouver [44,45]	Toronto [46]	Montreal [47,48,49]
Alert systems	BC Heat Alert Response System (HARS): Heat warning issued by Environment and Climate Change Canada and amplified by city, Vancouver Coastal Health, and provincial communications.	Harmonized Heat Warning and Information System (HWIS): Heat warning issued by Environment andClimate Change Canada and amplified by city communications.	Alerts released by the Direction Régionale de Santé Publique through the regional health system emergency coordination unit. Aligned across all government actors in the region.
Health information	Public messaging of heat health information and cooling station availability.	Public messaging of heat health information and service availability.	Public messaging of heat health information across public health, city, and community actors.
Cooling centres and services	Cooling centres activated within local public facilities. Outdoor heat respite, including shaded spaces, misting stations, and drinking fountains.	Heat relief network comprising > 600 public facilities (e.g., public libraries). Extended public pool hours. Measures to inform residents of heat relief strategies.	Air-conditioned buildings, pools, and water stations opened by the city and network.
Outreach to high-risk populations	Community outreach, including wellness checks and cool kit distribution by the City of Vancouver and community organizations. Funding opportunities for community organizations.	Street outreach for people experiencing homelessness by the City of Toronto and some community organizations.	Door-to-door check-ins conducted primarily by police officers and community organizations. Distribution of water to organizations serving unhoused populations.
Worker protections	Activate special measures to protect firefighters from heat impacts.	--	Activate special measures to protect city staff from heat impacts, such as firefighters.

**Table 3:** Formal documents designating heat response roles and responsibilities.

Governance Mechanism		Cases		
		Vancouver	Toronto	Montreal
Provincial Heat Alert Systems		BC HARS ^1^ [2024]	Ontario HWIS ^2^ [2023]	SUPREME ^3^
Heat Plans and Protocols		*Initial Response* *Guideline* [2020]	*Toronto Heat Relief* *Strategy* [2024]	*Plan particulier d’intervention—Chaleur extrême (Centre de sécurité civile)* City department protocols *Plan Régional de Prévention et de Protection: Chaleur Accablante et Extrême (Region of Montreal)* *Plan Particulier d’Intervention: Chaleur Accablante ou Extrême (local health units)* *Plan de Mesures d’Urgences—Chaleur Extrême (Urgences-santé)*
Mandates	Municipal	--	*Toronto Municipal Code, Chapter 59: Emergency* *Management*	*Plan de sécurité civile et de continuité des affaires de l’agglomération de Montréal* *[2010]*
	Provincial	*Public Health Act* [2008] *Emergency and Disaster Management Act* [2023]	*Ontario Public Health* *Standards* ^4^ [2023]	*Plan Ministériel de Gestion des Episodes* *de Chaleur Extrême [2021]* *Plan National de Sécurité Civile* *Loi sur les services préhospitaliers d’urgence [2002]* *Loi sur la Sante Publique [2001]* *Programme National de Santé Publique [2015]*

^1^ BC Heat Alert and Response System. ^2^ Harmonized Heat Warning and Information System. ^3^ Système de surveillance et de prévention des impacts sanitaires des événements météorologiques extrêmes. ^4^ The *Ontario Public Health Standards* do not function as a legislative mandate, but rather specify the minimum requirements for mandated local public health programs and services. Note: the titles of all formal documents are italicized.

**Table 4:** Formal collaborative governance structures and mechanisms.

Governance Mechanism		Cases		
		Vancouver	Toronto	Montreal
Coordinating structures	Municipal	Coordinating Calls	Hot Weather Response Coordinating Committee	City civil security coordinating centre
	Regional	Coordinating Calls	--	Health Mission—CRMUSCAR ^1^, Urgences-santé ^2^, DRSP ^3^, and regional communications (Ministry of Health and Social Services) Regional Organization for Civil Security (Ministry of Public Security)
	Provincial	BC HEAT Committee ^4^	--	Coordinating calls (Ministry of Health and Social Services)
Coordination meetings		Pre- and post-season; following activation	Pre- and post-season	Pre- and post-season; during activation
Agreements		--	--	*Entente de collaboration 2022–2030 entre la Ville de Montréal et la Direction régionale de santé publique* (Partnership between city and DRSP ^5^)

^1^ Coordination régionale des mesures d’urgence, sécurité civile et accès réseau. ^2^ Paramedic service. ^3^ Direction Régionale de la Santé Publique. ^4^ BC Health Effects of Anomalous Temperatures Committee. ^5^ Direction Régionale de la Santé Publique. Note: the titles of all formal documents are italicized.

## Data Availability

All data requests should be submitted to the corresponding author.

## Abbreviations

The following abbreviations are used in this manuscript:

BC	British Columbia
BC HARS	BC Heat Alert and Response System
BC HEAT Committee	BC Health Effects of Anomalous Temperatures Committee
CIUSSS	Centres intégrés universitaires de santé et de services sociaux
CRMUSCAR	Coordination régionale des mesures d’urgence, sécurité civile et accès réseau
DRSP	Direction Régionale de la Santé Publique
ECCC	Environment and Climate Change Canada
HWIS	Harmonized Heat Warning and Information System
SUPREME	Système de surveillance et de prévention des impacts sanitaires des événements météorologiques extrêmes
TEM	Toronto Emergency Management
TPH	Toronto Public Health
VCH	Vancouver Coastal Health Authority
VEMA	Vancouver Emergency Management Agency

## Appendix A. Interview Guide

1. Please describe your current role with [participant organization].
  - What is your specific role in [heat strategy]?
2. Please describe your organization’s role in [city] extreme heat strategy.
3. How does [city] heat strategy fit into your organization’s mandate?
4. How is your organization’s involvement with [city] heat strategy linked to climate-related strategies at the regional, provincial, or federal levels?
5. How and to what extent is equity factored into [city] heat strategy?
6. How is the implementation of [city] heat strategy monitored and evaluated?
7. Can you tell me about the nature and scope of collaborations your organization participates in to fulfil the objectives of [heat strategy]?
  - How were these collaborations initiated?
  - Nature of collaboration—e.g., formal and informal; core functions such as information sharing, joint planning; frequency of collaboration
  - How and by whom are roles and responsibilities assigned?
  - How and by whom are shared goals or objectives established?
  - How and by whom are decisions made?
  - How and to what extent does communication among collaborators typically occur?
  - How is progress toward stated goals and objectives monitored or evaluated?
  - How and by whom are conflicts or competing interests typically reconciled?
  - How have the collaborative arrangements you’ve described been documented or articulated?
8. Which actors or organizations are most influential in shaping the heat strategy and its implementation?
9. What role, if any, does public health play in the heat strategy?
  - To what extent does your organization collaborate with public health as part of the heat strategy?
10. Are there groups or organizations who are not currently involved with the strategy that you feel should be?
  - Why are they not currently involved?
  - What do you feel they might contribute?
11. Who oversees the governance of collaborative efforts?
  - What are the accountability mechanisms related to collaboration in place?
12. Based on your experience, what does effective collaboration to address extreme heat entail?
  - What facilitates effective collaboration?
  - What are the common or recurring barriers to effective collaboration?
13. Based on your experience, are there any lessons learned or recommendations you would like to share to inform the improvement of collaboration for addressing climate change impacts?
14. Are there any other insights or details related to [heat strategy] that you feel might enhance our understanding of its governance?

## Appendix B. Final Codebook

This codebook includes inductively added parent codes but excludes inductively added sub-codes, which varied by case.

**Table A1:** Codebook.

Parent Code	Sub-Codes
Roles and Responsibilities	
Relationships	
Mandates	Provincial
	Regional
	Local
Heat strategy description and approach	General
	Equity considerations
	Public health and preventative approach
	Community role
Link to climate strategies	
Evaluation and strategic updates	
Governance	Structure
	Nature/format
	Definition of roles
	Formalization
	Initiation
	Alignment of goals and values
	Communications
	Decision-making
	Conflict resolution
	Accountability mechanisms
	Expertise
	Level of trust
	Financing
	Number of participants
	Policy framework
	Power dynamics
Influential actors	
Missing actors (Actors not currently involved that participants suggest could play a role.)	
Challenges	
Facilitators and barriers to collaborative intersectoral response	
Suggestions for strengthening the strategy	
Miscellaneous	
